# Thalidomide-induced limb abnormalities in a humanized CYP3A mouse model

**DOI:** 10.1038/srep21419

**Published:** 2016-02-23

**Authors:** Yasuhiro Kazuki, Masaharu Akita, Kaoru Kobayashi, Mitsuhiko Osaki, Daisuke Satoh, Ryo Ohta, Satoshi Abe, Shoko Takehara, Kanako Kazuki, Hiroshi Yamazaki, Tetsuya Kamataki, Mitsuo Oshimura

**Affiliations:** 1 Department of Biomedical Science, Institute of Regenerative Medicine and Biofunction, Graduate School of Medical Science, Tottori University, 86 Nishi-cho, Yonago, Tottori 683-8503, Japan; 2 Chromosome Engineering Research Center (CERC), Tottori University, 86 Nishi-cho, Yonago, Tottori 683-8503, Japan; 3 Faculty of Family and Consumer Sciences, Department of Nutrition and Dietetics, Kamakura Women’s University, 6-1-3 Ofuna, Kamakura, Kanagawa 247-8512, Japan; 4 Graduate School of Pharmaceutical Sciences, Chiba University, 1-8-1 Inohana, Chuo-ku, Chiba 260-8675, Japan; 5 Division of Pathological Biochemistry, Department of Biomedical Sciences, Faculty of Medicine, Tottori University, 86 Nishi-cho, Yonago, Tottori 683-8503, Japan; 6 Division of Toxicology, Hatano Research Institute, Food and Drug Safety Center, 729-5 Ochiai, Hadano, Kanagawa 257-8523, Japan; 7 Showa Pharmaceutical University, 3-3165 Higashi-tamagawa Gakuen, Machida, Tokyo 194-8543, Japan; 8 Graduate School of Pharmaceutical Sciences, Hokkaido University, Nishi 6, Kita 12, Kita-ku, Sapporo 060-0812, Japan

## Abstract

Thalidomide is a teratogen in humans but not in rodents. It causes multiple birth defects including malformations of limbs, ears, and other organs. However, the species-specific mechanism of thalidomide teratogenicity is not completely understood. Reproduction of the human teratogenicity of thalidomide in rodents has previously failed because of the lack of a model reflecting human drug metabolism. In addition, because the maternal metabolic effect cannot be eliminated, the migration of unchanged thalidomide to embryos is suppressed, and the metabolic activation is insufficient to develop teratogenicity. Previously, we generated transchromosomic mice containing a human cytochrome P450 (CYP) 3A cluster in which the endogenous mouse *Cyp3a* genes were deleted. Here, we determined whether human CYP3A or mouse Cyp3a enzyme expression was related to the species difference in a whole embryo culture system using humanized CYP3A mouse embryos. Thalidomide-treated embryos with the human *CYP3A* gene cluster showed limb abnormalities, and human CYP3A was expressed in the placenta, suggesting that human CYP3A in the placenta may contribute to the teratogenicity of thalidomide. These data suggest that the humanized CYP3A mouse is a useful model to predict embryonic toxicity in humans.

Thalidomide has been previously used as a sedative and to treat morning sickness in pregnant women. However, infants with malformations characterized by limb hypoplasia were delivered of women who had taken thalidomide, and about 10,000 pregnant women experienced such incidents^1^,^2^,^3^,^4^. The failure to anticipate such incidents during the drug development phase is attributed to the species differences in thalidomide metabolism between humans and rodents^5^,^6^. Furthermore, no reports have identified the enzyme responsible for thalidomide teratogenicity in humans or rodents, and embryonic teratogenicity similar to that in humans has not been successfully reproduced in a rodent model.

Cytochrome P450 enzymes (CYPs) are responsible for the oxidative metabolism of a diverse range of xenobiotics^7^,^8^,^9^. Among human CYP family members, a human fetal specific form of CYP3A7 is detected as early as 50–60 days after the start of gestation and persists until after birth, when its abundance decreases over the first months of life^10^. Furthermore, CYP3A7 protein has been detected in the placenta^11^ and reported to mediate thalidomide metabolism^12^. Thus, CYP3A7 is assumed to be one of the enzymes related to drug-induced embryonic teratogenesis/procarcinogen activation^13^,^14^, although there is no direct evidence to support this assumption.

We believed that two issues prevented reproduction of embryonic teratogenicity in a rodent model. The first issue was the lack of a human metabolic enzyme-expressing animal that demonstrated human-specific teratogenicity at the embryonic stage. To address this issue, we generated transchromosomic (TC) mice containing a single copy of a 700 kb genomic region with *CYP3A4*, *CYP3A5*, *CYP3A7*, and *CYP3A43* genes, via a chromosome-engineering technique in which the endogenous mouse *Cyp3a* genes were deleted^15^. In this mouse strain with fully humanized *CYP3A* genes [designated as CYP3A-human artificial chromosome (HAC)/Cyp3a knock out (KO)] reproduced the kinetics of triazolam metabolism catalysed by CYP3A, CYP3A-mediated mechanism-based inactivation effects, and the formation of fetal-specific CYP3A7-mediated metabolites of dehydroepiandrosterone observed in humans. The second issue was that the unchanged drug could not avoid maternal metabolism to pass through the placenta and affect embryos because of the extremely high maternal metabolism of thalidomide in rodents compared with that in humans^6^. Thus, the unchanged drug did not affect embryos in rodents, explaining the failed reproduction of teratogenicity in a rodent model. To overcome the second issue, we employed whole-embryo culture (WEC) that enables evaluation of embryonic metabolism and toxicity without the interference of maternal metabolism^16^,^17^,^18^. In WEC, the whole body of an embryo including the placenta is removed from a dam and transferred into a culture vessel to observe embryonic developmental processes. In this study, we determined whether the human CYP3A or mouse Cyp3a enzymes were related to the species-specific teratogenicity in a WEC system using humanized CYP3A mouse embryos.

## Results and Discussion

To test whether human CYP3A or mouse Cyp3a enzymes were related to species-specific teratogenicity, thalidomide was administered to pregnant mice or added to culture medium of the WEC system. First, thalidomide was administered to pregnant Cyp3aKO female mice mated with CYP3A-HAC/Cyp3aKO male mice using a previously described method^19^. However, abnormalities were not detected in CYP3A-HAC-positive or -negative embryos compared with untreated controls following treatment (data not shown). This result might have been caused by the effects of the extensive maternal metabolism of thalidomide, which deactivates the drug in mice, as suggested previously^20^. In rodents, the metabolic capacity of thalidomide is substantially higher than in humans^6^. This results in markedly lower unchanged drug levels in maternal mouse blood than in human blood. This effect is also evident in chimeric mice transplanted with or without human hepatocytes after oral administration of thalidomide^21^. Because Cyp3aKO mice were used as pregnant mothers, drug-metabolizing enzymes other than mouse Cyp3a might be related to the extensive metabolism of thalidomide. Thus, a method was required to directly expose embryos to unchanged thalidomide without mediation of a maternal body.

The WEC system is an excellent method to screen drugs for teratogenic hazards^22^,^23^. Therefore, we used the WEC system to investigate the direct effects of thalidomide during the critical period of organogenesis in mouse embryos. In embryonic day 11.5 (E11.5) mouse embryos (*in vivo*, without culture), there were no differences in the crown–rump length, total number of somites, or total protein content of CYP3A-HAC-positive and -negative groups (Table 1). Embryos with abnormalities were not detected in either group. The body size of embryos at 24 h after culture in the WEC system was larger than that of E11.5 embryos (before culture) (Supplementary Fig. 1). Compared with the control group (CYP3A-HAC negative; vehicle), the number of embryonic heartbeats did not change during the culture period (Table 2). The number of heartbeats was maintained at about 200 beats/min. The crown–rump length, total number of somites, and total protein content of the whole embryo after culture were higher than those of E11.5 embryos (Tables 1 and 3). These data suggest that the embryos cultured in the WEC system grew in a similar manner to embryos *in utero*.

**Figure 1:**
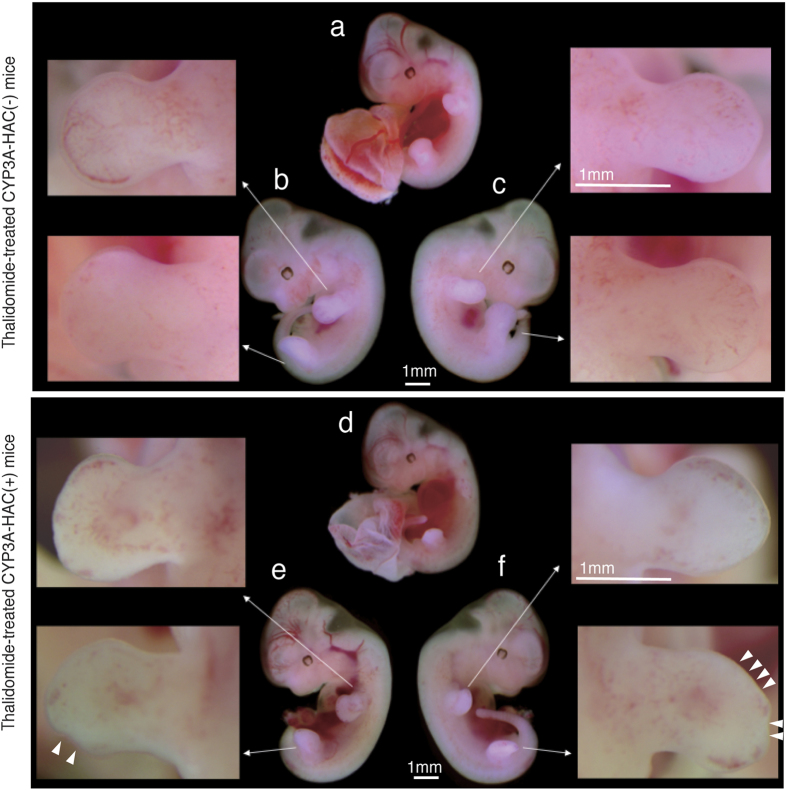
Morphological changes in cultured mouse embryos at 24 h after incubation with thalidomide. (**a**) Whole body of a CYP3A-human artificial chromosome (HAC)-negative mouse embryo with the placenta and yolk sac. The whole body of a CYP3A-HAC-negative mouse embryo without the placenta and yolk sac [left (**b**) and right (**c**) side]. (**d**) Whole body of a CYP3A-HAC-positive mouse embryo with the placenta and yolk sac. The whole body of a CYP3A-HAC-positive mouse embryo without the placenta and yolk sac [left (**e**) and right (**f**) side]. Arrows indicate an enlarged image of each limb. White triangles indicate a point of abnormality on the limb. Scale bar = 1 mm.

In addition, all groups showed no change in the crown–rump length, total number of somites, or total protein content after 24 h (Table 3). With respect to morphogenesis, no remarkable change was observed in cultured CYP3A-HAC-negative embryos treated with thalidomide after 24 h (Fig. 1a–c). However, limb abnormalities were observed in 42.9% (9/21) of cultured CYP3A-HAC-positive embryos treated with thalidomide, which was statistically significant compared with the other three groups (Table 3; Fig. 1d–f). In CYP3A-HAC-negative mouse embryos treated with thalidomide for 24 h, the limbs of cultured mouse embryos exhibited paddle-like hand or foot plates and the initial stages of digital ray formation. In contrast, 9 of 21 CYP3A-HAC-positive mouse embryos treated with thalidomide for 24 h formed limbs with bent hand or foot plates. The bent plates showed abnormalities but no morphogenetic delay. Among the nine abnormal embryos, a forelimb abnormality was observed in one embryo, a hindlimb abnormality was observed in seven embryos, and abnormalities in forelimbs and hindlimbs were observed in one embryo. Other morphological characteristics of CYP3A-HAC-positive embryos treated with thalidomide were unchanged compared with embryos in other groups. Additionally, limb abnormalities were not detected in thalidomide-treated embryos of wild-type mice such as ICR and C57BL (data not shown). The tip sides of the limbs are the sites that subsequently develop into the antebrachium and croup. For this reason, the morphogenetic abnormalities of the tip side of the limbs induced in the cultured embryos in this study were considered highly likely to lead to phocomelia, which is a typical malformation associated with thalidomide toxicity in humans^24^. Unfortunately, because of the limited morphogenesis of the limbs in the WEC system, it was unclear whether the morphogenetic abnormalities observed in the present study would eventually result in phocomelia. Moreover, the *in vivo* effects of thalidomide in the mice used in this study have not been confirmed as described above (data not shown).

**Figure 2:**
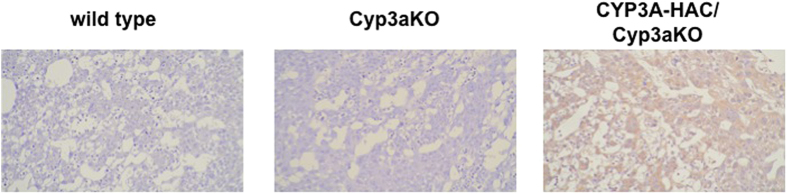
Immunohistochemical analyses of CYP3A in the placenta. Immunodetection of human CYP3A in wild-type (WT, left panel), Cyp3a knock out (KO) (middle panel), and CYP3A-HAC/Cyp3aKO (right panel) placentas. Original magnification: ×400.

To determine whether CYP3A protein was expressed in the placenta at E11.5, we performed expression analyses. Immunohistochemical analysis using an anti-CYP3A7 antibody revealed expression of human CYP3A in the placenta of CYP3A-HAC/Cyp3aKO mice, but CYP3A was not expressed in the placentas of wild-type or Cyp3aKO mice (Fig. 2). These data suggest that CYP3A from the humanized CYP3A placenta might mediate the metabolism of thalidomide.

In this study, the most remarkable finding is that abnormal embryos (i.e., those with limb abnormalities) were identified only in CYP3A-HAC-positive embryos treated with thalidomide in the WEC system. In humans, the affected children were delivered of women administrated with a p.o. dose as low as 100 mg thalidomide, i.e., about 2 mg/kg 25, and 0.9 μg/mL thalidomide in the circulating blood of pregnant women was sufficient to induce fetal abnormalities^26^. In this study, addition of 250 μg/mL thalidomide to the embryo culture medium induced limb abnormalities in CYP3A-HAC-positive embryos. However, we cannot directly compare the dose effect because of the differences of *in vivo* and *in vitro* experiments, and the administration methods. Various hypotheses for thalidomide activation have been proposed, including the generation of reactive oxygen species^27^, reactive acylating^28^ and arene oxide intermediates^29^, and inhibition of angiogenesis^30^ or the protein cereblon^31^. To the best of our knowledge, this is the first report indicating that human CYP3A may be related to thalidomide-induced teratogenicity. Although thalidomide metabolites generated by CYP3A derived from the placenta may be related to teratogenicity, the mechanism by which thalidomide induces limb abnormalities only in CYP3A-HAC/Cyp3aKO mouse embryos is unclear. Previous reports suggest that thalidomide is metabolized by two major pathways, 5′-hydroxythalidomide (a major product in rodents) and 5-hydroxythalidomide (human disproportionate phenyl ring-based metabolites)^12^,^21^. The second oxidation step in the human disproportionate 5-hydroxythalidomide pathway generates a reactive intermediate, possibly an arene oxide^29^, which can be trapped by glutathione (GSH) to produce GSH adducts and 5,6-dihydroxythalidomide^12^,^21^. These metabolites may be related to the limb abnormalities. In our preliminary study, placental preparations from one subject had detectable midazolam 1′-hydroxylation and thalidomide 5-hydroxylation activities that might have the potential for human CYP3A-dependent metabolic activation of thalidomide^12^,^21^. Future studies should focus on identification of thalidomide metabolites using embryonic tissues including embryo, yolk sac, and placenta. Additionally, studies should determine which thalidomide metabolites induce the abnormalities. In this study, we failed to show that thalidomide induced limb abnormalities under *in vivo* conditions. Nevertheless, our findings suggest that the CYP3A-HAC/Cyp3aKO mouse developed by TC technology may be a useful model not only to study the mechanism of thalidomide-induced limb abnormalities but also to assess embryonic toxicity of drugs in humans.

## Methods

### Genomic polymerase chain reaction (PCR) analyses

Genotyping of TC embryonic tissue was performed by genomic PCR analyses using human-specific *CYP3A* primers. Genomic DNA was extracted from the mouse embryonic yolk sac and placenta using a genomic extraction kit (Gentra System, Minneapolis, MN, USA), and PCR was performed after WEC. Primer pairs to detect the human *CYP3A* cluster were as follows: CYP3A4-4L/CYP3A4-3R (*CYP3A4*), 5′-TCCCCCTGAAATTAAGCTTA-3′ and 5′-TGAGGTCTCTGGTGTTCTCA-3′; CYP3A5F/CYP3A5R (*CYP3A5*), 5′-ATAGAAGGGTCTGTCTGGCTGG-3′ and 5′-TCAGCTGTGTGCTGTTGTTTGC-3′; and CYP3A7-3L/CYP3A7-3R (*CYP3A7*), 5′-TCCCCCTGAAATTACGCTTT-3′ and 5′-CATTTCAGGGTTCTATTTGT-3′. C57BL/6 and ICR mouse genomic DNA was used as negative controls.

### Ethics statement

All experimental procedures were approved by the Institutional Animal Care and Use Committee of Tottori University and Kamakura Women’s University. Methods were carried out in accordance with the approved guidelines of the Institutional Animal Care and Use Committee of Tottori University and Kamakura Women’s University.

### WEC

The WEC system (Model: 10-1-0310; Ikemoto Rika, Tokyo, Japan) used in this study has been described by New and Cockroft^22^,^23^. Compared with *in vivo* growth of embryos, the growth of cultured embryos is reported to decrease during culture^17^. To determine the culture stage and time in this study, we considered the period required for the effects of thalidomide to manifest on the limbs as well as the *in vivo* limb formation speed, and selected the optimal conditions based on the time during which embryo culture is possible. Thus, mouse embryos (E11.5) were cultured for 24 h in 100% rat serum (Institute for Animal Reproduction, Kasumigaura, Japan). After 2 h of incubation, thalidomide dissolved in dimethyl sulfoxide (Wako Pure Chemical Industries, Ltd, Osaka, Japan) was added to the medium (final concentration: 250 μg/mL) until the end of culture. The heartbeats of embryos were measured at 2, 4, and 24 h after culture. Embryos were examined for morphology, crown–rump length, total number of somites, and total protein content after culture for 24 h. Total protein contents were measured by the Lowry method as described previously^32^.

### Immunohistochemical analyses

All specimens were fixed with 10% formalin and embedded in paraffin. Paraffin-embedded 4 μm sections were dewaxed with xylene and then rehydrated gradually. Endogenous peroxidase activity was blocked by immersing the sections in 0.3% hydrogen peroxide in methanol for 30 min. After rinsing with PBS(−), the sections were immunostained using a Histofine Mouse Stain Kit (Nichirei, Tokyo, Japan). The primary antibody used in this study was an anti-CYP3A7 mouse monoclonal antibody (clone F19 P2 H2, 1:50, Abcam, Cambridge, UK). Immunoreactions were visualized with diaminobenzidine. The sections were counterstained with haematoxylin.

### Statistical analyses

Statistical analyses were performed with Fisher’s exact test for CYP3A-HAC-positive embryos treated with thalidomide compared with the other three groups.

## Additional Information

**How to cite this article**: Kazuki, Y. *et al.* Thalidomide-induced limb abnormalities in a humanized CYP3A mouse model. *Sci. Rep.* **6**, 21419; doi: 10.1038/srep21419 (2016).

## Supplementary Material

Supplementary Information

## Figures and Tables

**Table 1:** Growth and differentiation of mouse E11.5 embryos (without culture).

CYP3A-HAC	n	Crown-rumplength (mm)	Total numberof somites(No.)	Total proteincontents (μg/embryo)	Number of embryoswith abnormalities
—	10	7.3 ± 0.5	58.0 ± 3.5	1870 ± 403	0 (0%)
+	4	7.5 ± 0.3	59.8 ± 1.9	1975 ± 396	0 (0%)

mean ± S.D.

**Table 2:** Number of heartbeats of cultured mouse embryos over a 24 h incubation.

CYP3A-HAC	Thalidomide	n	Culture time (h)		
			2	4	24
−	−	40	194 ± 6.2	197 ± 8.4	201 ± 7.3
−	+	56	195 ± 5.6	196 ± 4.5	200 ± 8.6
+	−	12	195 ± 4.5	196 ± 4.8	201 ± 5.0
+	+	21	195 ± 4.0	195 ± 6.2	205 ± 9.7

Mouse E11.5 embryos were cultured for 24 h. (beats/min).

mean ± S.D.

**Table 3:** Effects of thalidomide on cultured embryos 24 h after incubation.

CYP3A-HAC	Thalidomide	n	Crown-rumplength (mm)	Total number ofsomites (No.)	Total proteincontents (μg/embryo)	Number of embryoswith abnormalities
−	−	40	8.8 ± 0.5	69.3 ± 3.2	2778 ± 557	0 (0%)
−	+	56	8.8 ± 0.6	67.5 ± 3.6	2821 ± 534	0 (0%)
+	−	12	8.8 ± 0.5	71.2 ± 1.5	2838 ± 658	0 (0%)
+	+	21	8.9 ± 0.5	68.2 ± 2.8	2708 ± 671	9 (42.9%)***

Mouse E11.5 embryos were cultured for 24 h.

mean ± S.D.

***0.001 > p (Fisher’s exact test).
