# Supplementary material for: Thalidomide-induced limb abnormalities in a humanized CYP3A mouse model
Source: Sci Rep. 2016 Feb 23;6:21419. doi: 10.1038/srep21419 (PMC4763305; doi:10.1038/srep21419)
Supplement: Supplementary Information [file srep21419-s1.pdf]

Supplementary information

Thalidomide-induced limb abnormalities in a humanized CYP3A mouse model

Yasuhiro Kazuki[1,2*], Masaharu Akita[3], Kaoru Kobayashi[4], Mitsuhiko Osaki[2,5], Daisuke Satoh[2], Ryo Ohta[6], Satoshi Abe[1], Shoko Takehara[2], Kanako Kazuki[2], Hiroshi Yamazaki[7], Tetsuya Kamataki[8], and Mitsuo Oshimura[2*]

[1]Department of Biomedical Science, Institute of Regenerative Medicine and Biofunction, Graduate School of Medical Science, Tottori University, 86 Nishi-cho, Yonago, Tottori 683-8503, Japan

[2]Chromosome Engineering Research Center (CERC), Tottori University, 86 Nishi-cho, Yonago, Tottori 683-8503, Japan

[3]Faculty of Family and Consumer Sciences, Department of Nutrition and Dietetics, Kamakura Women's University, 6-1-3 Ofuna, Kamakura, Kanagawa 247-8512, Japan

[4]Graduate School of Pharmaceutical Sciences, Chiba University, 1-8-1 Inohana, Chuo-ku, Chiba 260-8675, Japan

[5]Division of Pathological Biochemistry, Department of Biomedical Sciences, Faculty of Medicine, Tottori University, 86 Nishi-cho, Yonago, Tottori 683-8503, Japan

[6]Division of Toxicology, Hatano Research Institute, Food and Drug Safety Center, 729-5 Ochiai, Hadano, Kanagawa 257-8523, Japan

[7]Showa Pharmaceutical University, 3-3165 Higashi-tamagawa Gakuen, Machida, Tokyo 194-8543, Japan

[8]Graduate School of Pharmaceutical Sciences, Hokkaido University, Nishi 6, Kita 12,

Kita-ku, Sapporo 060-0812, Japan

*Correspondence should be addressed to Y.K. (kazuki@grape.med.tottori-u.ac.jp) or M.O. (oshimura@grape.med.tottori-u.ac.jp). Department of Biomedical Science, Institute of Regenerative Medicine and Biofunction, Graduate School of Medical Science, Tottori University, 86 Nishi-cho, Yonago, Tottori 683-8503, Japan. Tel: +81-859-38-6219. Fax: +81-859-38-6210.

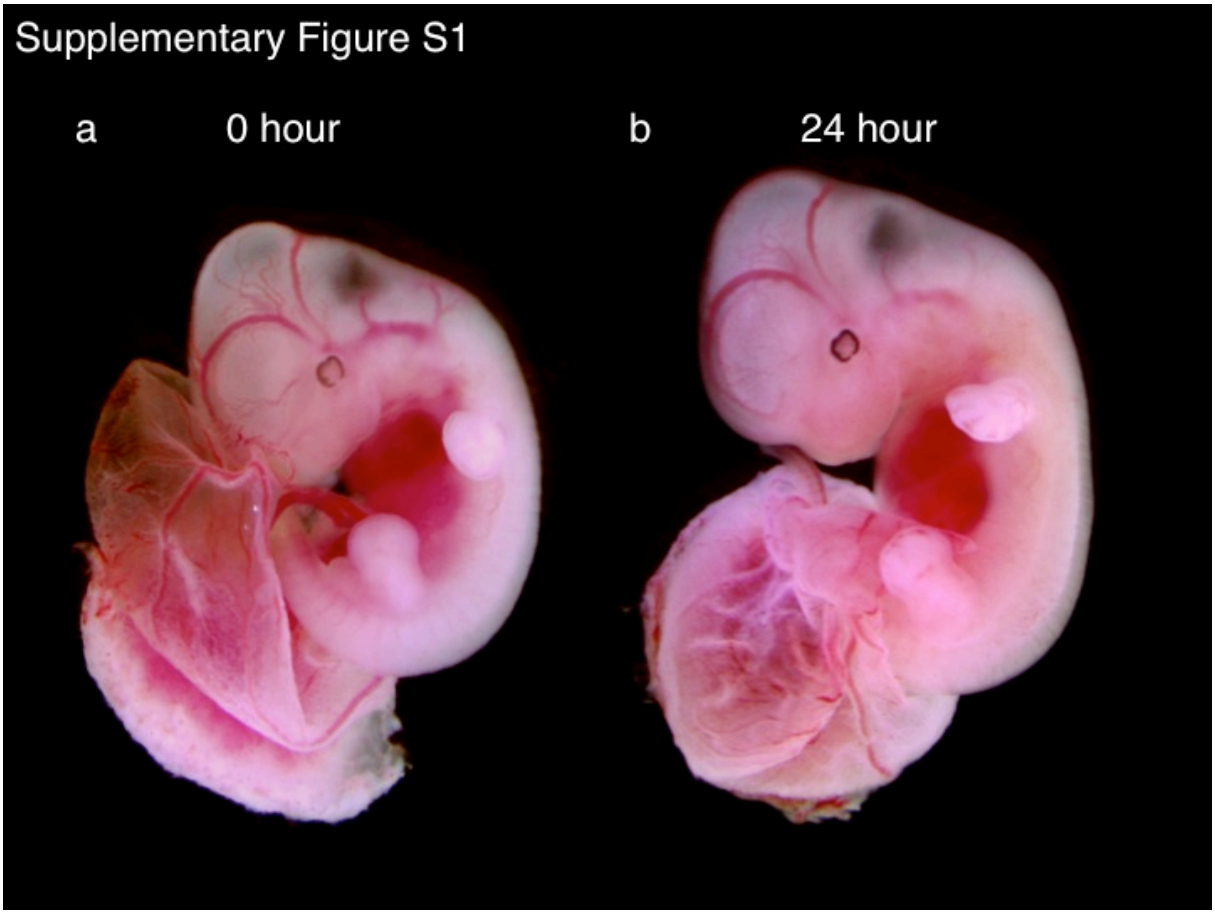

Supplementary Figure S1. Time course of normal morphological changes in cultured mouse embryos.

(a) The embryo immediately after being isolated from the uterus (incubation time = 0 h; embryonic day 11.5). (b) The embryo after incubation for 24 h.
